# Circulating miRNA signatures of early pregnancy in cattle

**DOI:** 10.1186/s12864-016-2529-1

**Published:** 2016-03-03

**Authors:** Jason Ioannidis, F. Xavier Donadeu

**Affiliations:** The Roslin Institute and R(D)SVS, University of Edinburgh, Easter Bush, Midlothian, EH25 9RG Scotland UK

**Keywords:** microRNA, Circulating, Biomarker, Early pregnancy, Bovine, miRNA sequencing, PCR array

## Abstract

**Background:**

Low fertility remains a leading cause of poor productivity in dairy cattle. In this context, there is significant interest in developing novel tools for accurate early diagnosis of pregnancy. MicroRNAs (miRNAs) are short RNA molecules which are critically involved in regulating gene expression during both health and disease. MiRNAs have been shown to regulate ovarian function, uterine receptivity, embryonic development and placental function. Circulating miRNAs can provide useful biomarkers of tissue function and disease; importantly, differential miRNA profiles have been linked to pregnancy and preeclampsia in humans. This study sought to establish the potential of circulating miRNAs as biomarkers of early pregnancy in cattle.

**Results:**

We applied Illumina small-RNA sequencing to profile miRNAs in plasma samples collected from eight non-pregnant heifers on Days 0, 8 and 16 of the oestrous cycle and 11 heifers on Days 16 and 24 of pregnancy. We sequenced a total of 46 samples and generated 9.2 million miRNA reads per sample. There were no differences in miRNA read abundance between any of the pregnant and non-pregnant time-points (FDR > 0.1). As a complementary approach, we analysed sample pools (3–4 samples/pool) corresponding to Days 0, 8 and 16 of the oestrous cycle and Day 24 of pregnancy (*n* = 3 pools/group) using Qiagen PCR arrays. A total of 16 miRNAs were differentially expressed (FDR < 0.1) in plasma between pregnant and non-pregnant animals. RT-qPCR validation using the same plasma samples confirmed that miR-26a was differentially upregulated on Day 16 pregnant relative to non-pregnant heifers (1.7-fold; *P* = 0.043), whereas miR-1249 tended to be upregulated in Day 16 pregnant heifers (1.6-fold; *P* = 0.081). Further validation in an independent group of heifers confirmed an increase in plasma miR-26a levels during early pregnancy, which was significant only on Day 24 (2.0-fold; *P* = 0.027).

**Conclusions:**

Through genome-wide analyses we have successfully profiled plasma miRNA populations associated with early pregnancy in cattle. We have identified miR-26a as a potential circulating biomarker of early pregnancy.

**Electronic supplementary material:**

The online version of this article (doi:10.1186/s12864-016-2529-1) contains supplementary material, which is available to authorized users.

## Background

As of December 2014, there were approximately 9.7 million cattle in the UK [1]. It is estimated that low fertility and suboptimal management of dairy herds cost £500 million in lost productivity annually [2, 3]. This problem is currently maintained by the chronic selection and use of high-yielding dairy cows. Profitable milk production depends on regular calving, the target being that every cow produces one calf a year. An important limitation to meeting this target is that, although conception rates remain very low (less than 50 % at first service), tools for accurate early detection of pregnancy (within 3 weeks) are not available leading to prolonged inter-calving intervals and significant losses in milk production. In light of this, the veterinary and dairy sectors have a particular interest in the development of novel biomarkers of early pregnancy.

MicroRNAs (miRNAs) have recently emerged as promising diagnostic biomarkers with high clinical potential. MiRNAs are short, non-coding RNA molecules which are centrally involved in post-transcriptional control of gene expression [4]. Different roles of miRNAs in the reproductive system have been proposed including the development of ovarian follicles and the corpus luteum [5, 6], uterine cyclicity and establishment of pregnancy, and embryonic development [7–10]. In the early developing bovine conceptus, the levels of some miRNAs including miR-496 and miR-125a vary greatly suggesting a role in the maternal-to-zygotic transcriptional transition [11]. Furthermore, several miRNAs including miR-27a and miR-92b are differentially expressed during the development of the placenta, where they have been associated with trophoblast differentiation and vascularisation [12, 13]. Finally, let-7 and miR-125b among other miRNAs have been shown to control mammary gland development and lactation [14].

MiRNAs are naturally secreted from cells into body fluids where they remain in relatively stable protein or lipid complexes and can be easily quantified [15–18]. This, combined with the fact that some miRNAs are tissue or developmental stage specific presents the opportunity to use miRNAs as non-invasive biomarkers of tissue function associated with a variety of physiological states (e.g. pregnancy) and diseases (e.g. neoplasia, cardiovascular disease, osteoarthritis, sepsis) [19, 20]. Indeed, miRNA-based platforms are currently being used for clinical diagnosis of various types of human cancer [19, 20]. Despite this, there are still limitations associated with measurement of circulating miRNA levels using existing technologies, which are derived from the presence of enzymatic inhibitors in serum and plasma, the low RNA content in bio-fluids, haemolysis and other cell contamination, and the need for unbiased procedures for normalisation of miRNA expression data [15, 21].

There is promising evidence of the potential of miRNAs as biomarkers of pregnancy. The circulating levels of miRNAs belonging to the primate- and placenta-specific C19MC cluster increase with gestational age, while levels of C14MC cluster miRNAs increase in the first trimester and decrease in later pregnancy [22, 23]. Furthermore, the C19MC cluster miRNAs, miR-516-5p, miR-518b, miR-520a and miR-525, are detectable in the human maternal circulation as early as 12 weeks of gestation [24]. In addition, the circulating levels of some C19MC miRNAs are significantly correlated with placental weight [25], consistent with their secretion from the developing placenta. Recent studies in humans have also identified miR-141 and miR-149 as pregnancy-associated circulating miRNAs; circulating levels of miR-141 significantly increase during gestation whereas the levels of both miRNAs decrease after delivery [26]. In sheep, miR-30c, miR-132, miR-379, miR-199a-3p and miR-320 are differentially expressed in serum on Days 30 or 60 of pregnancy [27]. There is limited information on pregnancy-related miRNAs in livestock and, to our knowledge, the levels of circulating miRNAs during early pregnancy have not been previously published for any domestic species.

The aim of this study was to profile miRNA levels in the plasma of cattle during Days 16 and 24 of pregnancy and to identify miRNA signatures that could be potentially used for diagnosis of early pregnancy. We present miRNA profiling results generated using two independent approaches, Illumina small-RNA sequencing and Qiagen PCR array.

## Results and Discussion

### Optimisation of bovine plasma miRNA profiling

We deemed it important to introduce sample quality control measures and optimise our quantification methodology before proceeding with profiling miRNAs in the bovine circulation, in order to address common problems in circulating miRNA quantification which might bias our study, such as low RNA yields, enzymatic inhibition and haemolysis.

**Fig. 1:**
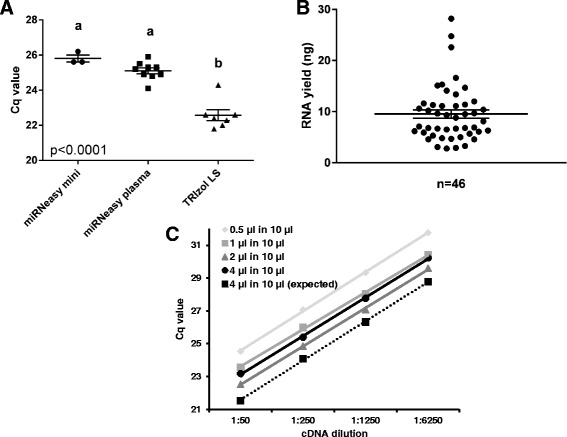
Optimisation of methodology for profiling miRNAs in bovine plasma. **a** RT-qPCR data plots (with mean ± SEM) showing abundance of the exogenous miRNA, cel-miR-39-3p, in plasma samples after RNA extraction using 3 different kits as indicated. **b** RNA yield from 1.05 mL bovine plasma samples using TRIzol LS. **c** Results of RT-qPCR quantification of miR-451 in plasma (mean ± SEM) using different RNA volumes for reverse-transcription; highest reaction efficiency was obtained using 2 μL of RNA in a 10 μL cDNA synthesis reaction

We tested three different commercial kits for extraction of RNA from bovine plasma, namely, miRNeasy mini (Qiagen, Netherlands), miRNeasy plasma (Qiagen), and TRIzol LS (Life Technologies, United Kingdom). Using 200 μL of bovine plasma, the TRIzol LS protocol was more efficient than the column-based kits as determined by RT-qPCR quantification of spiked-in exogenous cel-miR-39-3p (Fig. 1a). An added advantage of the TRIzol LS protocol is that it allows scaling-up the extraction volume; for our experiments we decided to use 1.05 mL of bovine plasma which yielded a mean of 9.5 ± 0.8 ng of RNA (Fig. 1b). This yield is similar to that reported for bovine plasma in another study (8.6 ng/mL), but lower than the mean yield reported for human plasma (25 ng/mL) [22, 28].

The presence of high levels of enzyme inhibitors in plasma samples (for example, immunoglobulin G) can significantly reduce the efficiency of RT-qPCR [29]. To address this, we tested different input RNA volumes and determined that using 2 μL of RNA extract in a 10 μL cDNA synthesis reaction yielded the highest reaction efficiency (Fig. 1c).

Another significant problem in plasma miRNA profiling is red blood cell contamination. As high levels of red blood cell-derived miRNAs such as miR-451 are associated with haemolysis [30, 31], the ΔCq between miR-451 and miR-23a has been proposed as a useful indicator of haemolysis [32]. We confirmed the validity of this approach for bovine samples by showing good correlation between the ratio of miR-451 and miR-23a and optical densities at 414 nm (Additional file 1, A-B). Based on this, we concluded that all samples used for miRNA profiling were within the normal range for non-haemolysed bovine plasma (Additional file 1, C-D).

### Illumina small-RNA sequencing of bovine plasma

We sequenced a total of 46 small RNA libraries generated from plasma samples collected from eight non-pregnant animals (on each of Days 0, 8 and 16 of the oestrous cycle), and 11 animals on each of Days 16 and 24 of pregnancy (encompassing the period for which a pregnancy biomarker would be desired [33]).

**Fig. 2:**
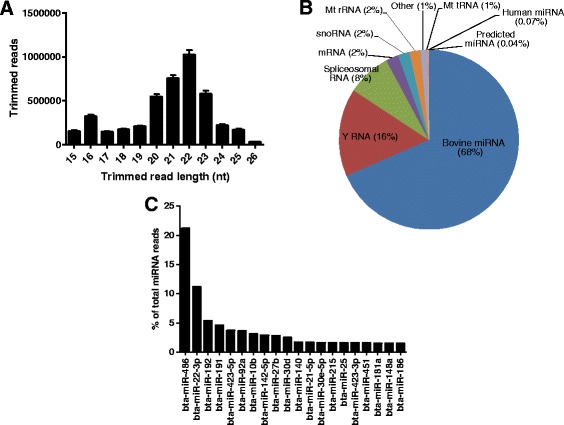
Summary of small-RNA sequencing read count data. **a** Length distribution (mean ± SEM) of read counts (after trimming) from Illumina sequencing of bovine plasma samples (nt = nucleotides). **b** Relative abundance of different RNA species sequenced in bovine plasma. **c** Top 20 miRNAs in bovine plasma detected by Illumina small-RNA sequencing; mean percentages of the total miRNA reads per million mapped (RPMMs) are shown

We obtained a median of 9.2 million raw sequencing reads from each sample (Table 1). A length distribution plot of reads post-trimming showed a distinct peak at 20–23 nucleotides indicating the majority of reads corresponded to mature miRNAs (Fig. 2a). After removal of low-quality reads, a median of 4 million reads (43.5 % of total reads) from each sample were mapped to the bovine genome, 68 % of which (2.7 million) were identified as miRNAs (Table 1). The remaining mapped reads corresponded to non-coding regulatory and structural small RNAs including Y-RNAs and spliceosomal RNAs, and fragments of larger RNA species such as mRNAs (Fig. 2b). The vast majority of miRNA reads (99.8 %) corresponded to registered bovine sequences in miRBase; the remaining corresponded to human miRNA homologues (0.11 %) or to predicted novel miRNAs (0.06 %, Fig. 2b). The percentage of miRNA reads obtained over the total sequencing reads (30.4 %) was higher than that reported from sequencing of bovine plasma in another study (5 %) but lower than obtained from human plasma using the same sequencing platform (57.7 %) [22, 34].

**Table 1:** Summary of results from small-RNA sequencing analyses

	NP	P16	P24	All
Total sequence reads	9.1 (8.8-9.6)	9.4 (8.7–11.6)	9.9 (8.8–10.7)	9.2 (8.7–10.5)
Reads with adapter	8.0 (7.7–8.9)	8.7 (7.7–10.6)	9.3 (8.0–9.8)	8.3 (7.7–9.6)
Reads which passed QC	4.5 (3.5–5.4)	4.0 (3.3–5.3)	4.0 (2.9–5.6)	4.2 (3.4–5.4)
Reads mapping to genome	4.3 (3.3–5.2)	3.7 (3.1–4.9)	3.6 (2.6–5.4)	4.0 (3.2–5.2)
Total miRNA reads	3.2 (2.2–3.8)	2.2 (2.1–3.0)	2.6 (1.5–3.5)	2.8 (2.1–3.5)

Median (in millions) and 25 % - 75 % percentile (in brackets) values obtained from a total of 46 plasma samples from non-pregnant heifers (NP; Days 0, 8 and 16 after oestrus combined; *n* = 24) and heifers on Days 16 (P16) and 24 (P24) of pregnancy (*n* = 11)

Overall, a total of 386 unique miRNAs across all individual samples (328 bovine, 37 human and 21 predicted novel) had more than 10 read counts. On average, the 10 most abundant miRNAs accounted for 61 % of the total miRNA reads in each sample (Fig. 2c). Out of the most abundant miRNAs in bovine plasma, miR-486 and miR-92a are reportedly expressed primarily in erythrocytes, and miR-191 is expressed primarily in platelets [31, 35]. Almost half (40 %) of the 10 most abundant miRNAs were also identified as highly abundant in bovine plasma in another study whereas 20 % were very abundant in human plasma [22, 28]. Common miRNAs identified across studies include miR-486, miR-92a, miR-192 and miR-423-5p.

**Fig. 3:**
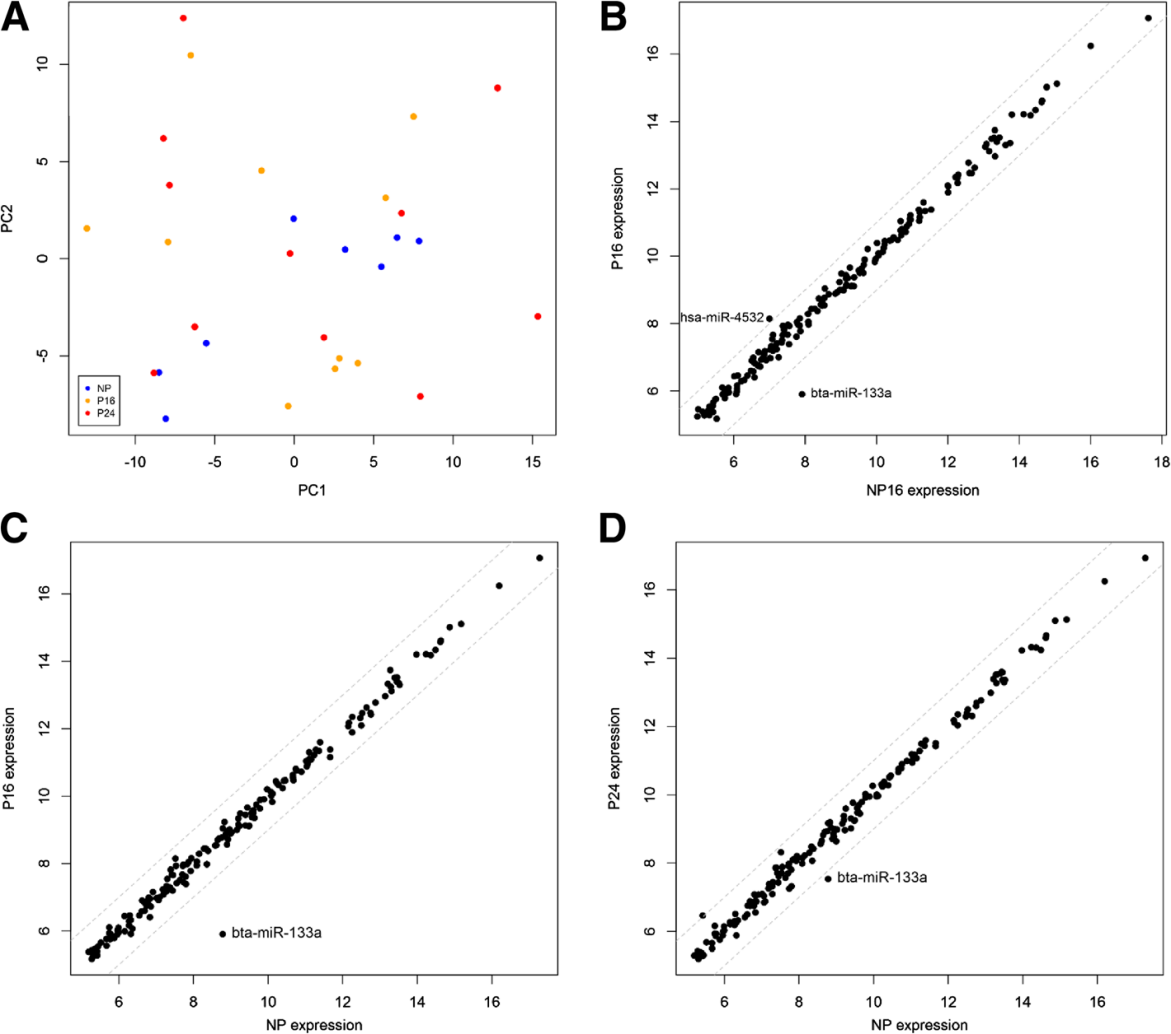
Results of differential expression analysis of miRNA sequencing data. **a** PCA plot using data from all pregnant (P16 and P24) and non-pregnant (NP) time-points obtained by Illumina small-RNA sequencing. Scatterplots for P16 vs NP16 (**b**) P16 vs NP (**c**) and P24 vs NP (**d**) comparisons using individual miRNA data are shown. The grey dotted lines indicate the 2-fold change threshold, and miRNAs which changed more than 2-fold between groups have been labelled. All data have been log2 transformed

One sample from Day 16 of pregnancy was removed prior to differential expression analysis because it distinctly had very low total reads per million mapped (RPMMs). Different comparisons of miRNA expression data between non-pregnant (NP) and pregnant groups (P) were made involving 1) the average expression over Days 0, 8 and 16 of the oestrous cycle (NP; *n* = 8) for the non-pregnant group vs each of Day 16 and Day 24 of pregnancy (P16 and P24; *n* = 10 and 11, respectively) and 2) a direct comparison between Day 16 of the oestrus cycle (NP16) and Day 16 of pregnancy. Principal component analysis based on these comparisons revealed no clear separation according to pregnancy status (Fig. 3a). Changes in the levels of 178 individual miRNAs which passed our quality filters (see Methods) were determined for each comparison (Additional file 2). Differences in the expression of a limited number of individual miRNAs were detected although they were generally small (under 2.5-fold) and not significant after FDR adjustment (Fig. 3b-d; Table 2). For one of the miRNAs, miR-133a, levels were up to 7.4-fold lower in Day 16 pregnant relative to non-pregnant heifers (FDR = 0.127) although the miRNA was not detectable by qPCR and thus those differences could not be validated further.

**Table 2:** Changes in circulating miRNA abundance identified by small RNA sequencing

	Comparison	Fold change	*P* value
bta-miR-101	NP16 vs P16	0.78	0.045
bta-miR-25	NP16 vs P16	0.81	0.014
bta-miR-19a	NP16 vs P16	1.22	0.029
bta-miR-130b	NP16 vs P16	1.33	0.019
bta-miR-328	NP16 vs P16	1.34	0.006
bta-miR-301b	NP16 vs P16	1.49	0.045
hsa-miR-4532	NP16 vs P16	2.21	0.006
bta-miR-133a	NP vs P16	0.14	0.001
bta-miR-193b	NP vs P16	0.74	0.021
bta-miR-365-3p	NP vs P16	0.75	0.026
bta-miR-2957	NP vs P16	0.77	0.048
bta-miR-152	NP vs P16	0.77	0.048
bta-miR-107	NP vs P16	0.82	0.036
bta-miR-103	NP vs P16	0.83	0.037
bta-miR-328	NP vs P16	1.22	0.003
bta-miR-133a	NP vs P24	0.42	0.028
bta-miR-125b	NP vs P24	0.71	0.024
bta-miR-99a-5p	NP vs P24	0.72	0.016
bta-miR-365-3p	NP vs P24	0.74	0.013
bta-miR-99b	NP vs P24	0.77	0.022
bta-miR-10b	NP vs P24	0.84	0.037

List of plasma miRNAs with different abundance between pregnant and non-pregnant heifers (NP16 vs P16, NP vs P16 and NP vs P24 comparisons) based on RPMM values from Illumina sequencing. Fold change in pregnant relative to non-pregnant heifers are shown. FDR > 0.1 for all miRNAs

### Bovine plasma miRNA profiling using Qiagen PCR arrays

To complement our sequencing analyses we used a commercial Custom PCR array platform to profile the expression of 308 unique bovine miRNAs in the same plasma samples. As it is not feasible to screen a large number of samples using PCR arrays, we analysed 3 sample pools (3–4 samples/pool) from each of Days 0, 8 and 16 from non-pregnant heifers and from Day 24 pregnant heifers (we reasoned we would more easily find differences in miRNA expression at Day 24 than at Day 16 of pregnancy).

**Fig. 4:**
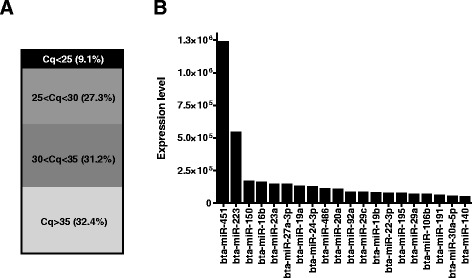
miRNA abundance in bovine plasma pools determined by Qiagen PCR arrays. **a** Distribution of mean Cq values for 308 conserved miRNAs. **b** Expression level of the 20 most abundant miRNAs in bovine plasma (calculated as 2^^(40-Ct)^)

We detected a total of 208 miRNAs in bovine plasma (based on mean Cq < 35 across all sample pools; Fig. 4a), the most abundant of which (Fig. 4b) corresponded to miRNAs reportedly expressed at high levels in blood cells including erythrocytes (miR-451, miR-486, miR-16), leukocytes (miR-150, miR-27a, miR-23a) and thrombocytes (miR-223, miR-20a, miR-24), and which are putatively released into the plasma through apoptosis, lysis or active secretion [36–38]. The absence of detectable haemolysis in our samples (see above) indicates the detected blood cell miRNAs are predominantly the result of secretion/activation and not cell lysis.

Comparing the 20 most abundant miRNAs in each of the PCR array and sequencing datasets, only 6 miRNAs (miR-486, miR-22-3p, miR-191, miR-92a, miR-140 and miR-451) were common. A very abundant miRNA in the sequencing dataset (miR-21-5p) could not be compared as it was not included in the PCR array. The overall poor agreement in abundant miRNAs identified by the two techniques can in part be explained by platform-specific biases such as those associated with sequencing adaptor ligation and differences in primer-specific PCR efficiency, which are known to affect the representation of miRNAs in a library therefore changing the perceived order of abundance of the miRNAs within a sample [39–41].

**Fig. 5:**
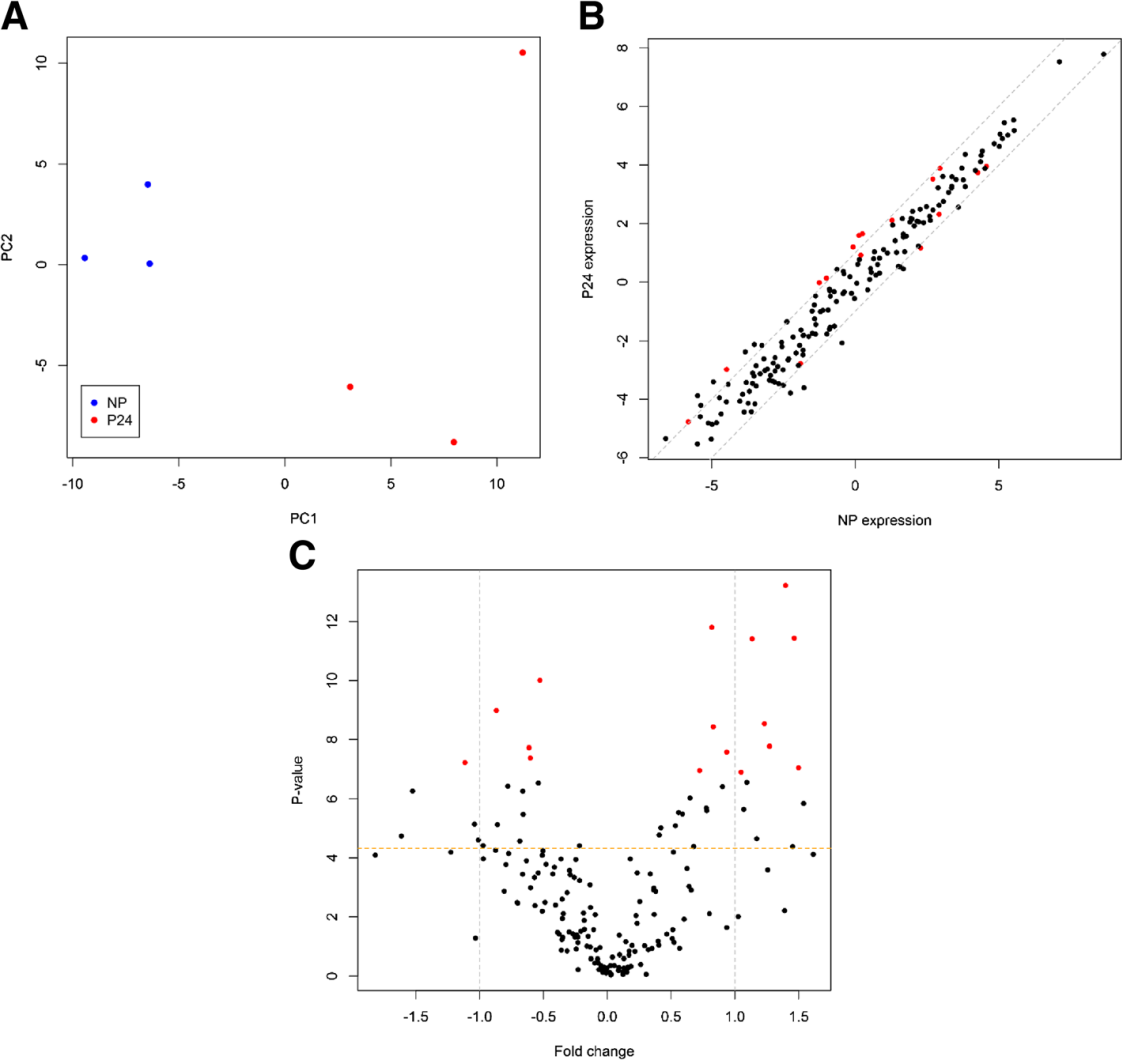
Differential expression analysis using PCR array data. **a** PCA plot and **b** scatterplot of individual miRNA data (NP vs P24). The grey dotted lines in **b**) indicate the 2-fold change threshold, and the highlighted data-points (in red) represent FDR < 0.1. The data have been log2 transformed. **c** Volcano plot for the same data shown in (**b**). The horizontal dotted orange line indicates the significance threshold (*P* = 0.05) and the vertical grey lines indicate the 2-fold change threshold. Data-points representing differences with FDR < 0.1 have been highlighted in red. The data have been log2 transformed

As before, differential expression analyses for each miRNA was performed considering the average expression value over Days 0, 8 and 16 of the oestrous cycle for the non-pregnant group (NP). Principal component analysis of PCR array results showed a clear separation between NP and P24 groups (Fig. 5a). Of the 176 unique miRNAs included in the analysis (after excluding low abundance miRNAs; see Methods), a total of 16 miRNAs were differentially expressed between the two groups (FDR < 0.1), 8 of which at ≥ 2-fold (Fig. 5b-c; Table 3; Additional file 3).

**Table 3:** Differences in circulating miRNA abundance identified by PCR array analyses

	Fold change	*P* value	FDR
bta-miR-101	0.46	0.007	0.090
bta-miR-141	0.55	0.002	0.058
bta-miR-29c	0.65	0.005	0.083
bta-miR-339a	0.66	0.006	0.088
bta-miR-29a	0.69	0.001	0.034
bta-miR-103	1.65	0.008	0.092
bta-miR-26a	1.76	0.000	0.016
bta-miR-30b-5p	1.78	0.003	0.064
bta-miR-26b	1.91	0.005	0.083
bta-miR-631	2.07	0.008	0.092
bta-miR-374b	2.20	0.000	0.016
bta-miR-151-5p	2.35	0.003	0.064
bta-let-7d	2.42	0.005	0.083
bta-miR-30c	2.63	0.000	0.016
bta-let-7f	2.76	0.000	0.016
bta-miR-454	2.83	0.008	0.092

List of differentially expressed miRNAs on Day 24 of pregnancy (NP vs P24; FDR < 0.1) based on Qiagen PCR array expression data. Fold changes were calculated as pregnant/non-pregnant

Generally, we observed poor overlap between differentially expressed gene lists from the two high-throughput datasets. Thus, only four of all miRNAs differentially expressed (*P* < 0.05) between pregnant (Day 24) and non-pregnant animals in the PCR array, i.e. miR-99b, miR-152, miR-101, miR-103, were also different (*P* < 0.05) between pregnant and non-pregnant groups (all comparisons) in the sequencing dataset. In some cases, the poor overlap was due to the miRNAs being under-represented in the sequencing libraries (e.g. miR-29c, miR-1249) resulting in low-confidence profiles. Other miRNAs such as miR-4532 were not included in the PCR arrays, therefore cross-platform validation was not possible. Platform-specific biases (indicated above) and the fact that pooled rather than individual samples were used in the PCR arrays may have also contributed to the relative lack of agreement between sequencing and PCR arrays. Taken together, our results highlight the limitations of current circulating miRNA profiling technologies, especially when subtle differences in gene expression are investigated, such as here, and show the importance of cross-platform validation.

### RT-qPCR validation of high-throughput data

From the results of PCR array and small-RNA sequencing we selected (based on both fold-change and P value) a total of 21 differences in miRNA abundance (involving 17 unique miRNAs) for validation by RT-qPCR (Additional file 4). Since we did not want our selection of miRNA candidates to be constrained by the limited number of differences originally obtained after FDR adjustment (FDR < 0.1; Tables 2 and 3), in the validation analyses we included 13 differences which were significant (*P* < 0.05) only before FDR adjustment. Because Day 16 of pregnancy was not included in the PCR array analyses (from where a majority of differences for validation were obtained), for the sake of inclusiveness both P16 and P24 were included in all validation analyses.

**Fig. 6:**
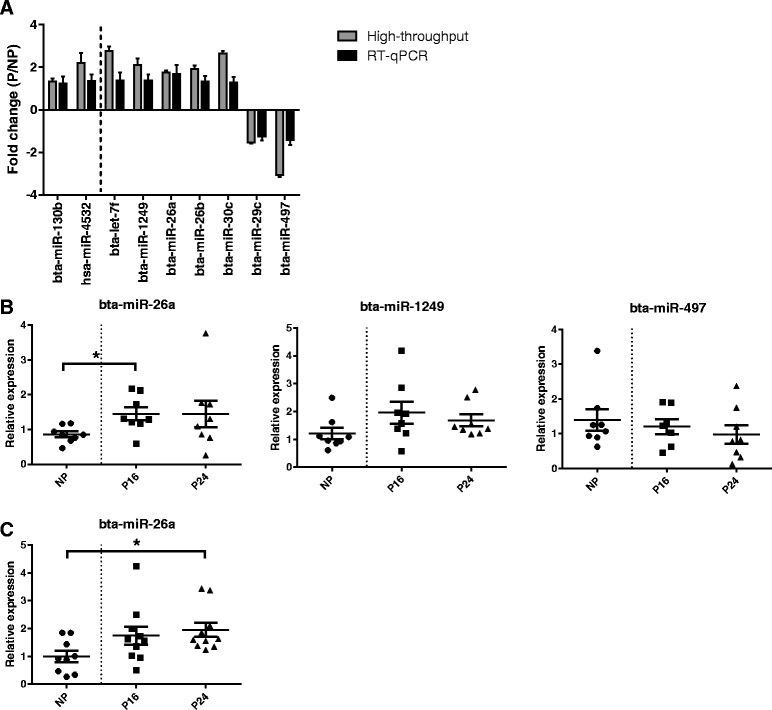
Results of RT-qPCR validation of high-throughput analyses data. **a** Comparative fold-changes (mean ± SEM) in miRNA abundance between pregnant and non-pregnant groups obtained by high-throughput analyses (PCR array or sequencing) and RT-qPCR. Results to the left and right of the dotted line correspond to NP16 vs P16 and NP vs P24 comparisons, respectively (Additional file 4). **b** RT-qPCR data plots (with mean ± SEM) for selected comparisons between pregnant and non-pregnant groups including the two differences in miRNA abundance that were significant (miR-26a, * indicates *P* < 0.05) or tended to be significant (miR-1249, NP vs P16, *P* < 0.1). **c** RT-qPCR data plots (with mean ± SEM) obtained from an independent group of heifers and which confirms an increase in plasma miR-26a levels during early pregnancy. NP corresponds to plasma samples collected before insemination (Day 0) from the same animals

We could robustly quantify 15 individual miRNAs by RT-qPCR representing a total of 17 differences in miRNA abundance between pregnant and non-pregnant groups. For 9 of these differences (Fig. 6a) the results of qPCR were consistent with those obtained by PCR array or sequencing although significance was only obtained for miR-26a; an increase in the levels of this miRNA was significant on Day 16 (NP vs P16, 1.7 fold, *P* = 0.043) but not on Day 24 (1.7 fold, *P* = 0.208 Fig. 6b). A trend for an increase in miR-1249 levels on Day 16 of pregnancy was also identified but was not statistically significant (NP vs P16, *P* = 0.081; Fig. 6b). To more robustly validate the changes in miR-26a during early pregnancy, particularly as they were relatively small, we analysed plasma samples from a different, larger group of heifers during early pregnancy (Fig. 6c). In this group of animals, when compared to levels before insemination, plasma levels of miR-26a were higher on Day 24 of pregnancy (2.0-fold; *P* = 0.027). On average, miR-26a levels were also higher on Day 16 (1.7-fold) although this difference did not reach significance (*P* = 0.118). Thus, from analyses in two independent groups of animals we concluded that the levels of miR-26a increase during early pregnancy in heifers.

Although expressed ubiquitously, distinctly high levels of miR-26a have been reported in the embryo, the ovary, and immune-related tissues and cells such as the thymus and B/T cells of different species [42–45]. Interestingly, miR-26a has been shown to directly down-regulate IFN-β, a major cytokine involved in innate and adaptive immune responses, suggesting a potential role for this miRNA as an immuno-suppressor during early pregnancy [46]. Additionally, recent studies in pig and goat have reported increasing levels of miR-26a in the conceptus and the ovary as early as Day 20 of pregnancy [47, 48]. Also, high plasma miR-26a levels have been associated with pre-eclampsia in humans [49]. These findings support the notion that miR-26a is involved in pregnancy, possibly by exerting immunomodulatory effects. Further studies should investigate the precise origin and function of this miRNA during pregnancy.

Mir-1249, identified by PCR array analyses as differentially expressed in pregnant animals, was severely under-represented in our sequencing library, with only 6.1 counts per million on average, which prevented any conclusions about its expression in bovine plasma. This may be the result of sequence- and secondary structure-dependent ligation biases during library preparation, a common issue with next-generation sequencing technology which affects all samples equally and thus is not considered to bias differential expression analyses [39–41]. The case of miR-1249 is a good example of the need to use different quantification methods for cross-platform validation of results. The levels of miR-1249 in granulosa cells have been shown to vary during follicle development in bovine [50], and miR-1249 expression has been confirmed in bovine mammary glands, however little is known about its expression and functions in other tissues [51].

The fact that results of RT-qPCR and high-throughput analyses agreed (i.e. differences between pregnant and non-pregnant groups showed the same trend) for only 9 of the 17 differences in miRNA abundance analysed may have been due in part to the intrinsically very low levels of RNA contained in plasma; this, together with the presence of several inhibitors of RT-qPCR enzymes in plasma make accurate quantification particularly challenging, especially when differences between the experimental groups being compared are small. Added to these are platform-specific biases and intrinsic inter-animal variation. In the case of the PCR arrays, the use of pooled samples may also have contributed to disagreement in the results from different profiling platforms. A recent study reported a 54.6 % agreement in human liver and brain tissue miRNA profiles across RT-qPCR, microarrays and small-RNA sequencing platforms [52], which is comparable to the agreement reported in our study. This most likely reflects the limitations associated with quantification of plasma miRNAs discussed above. Finally, we only attempted to validate a fraction of the differences identified in the PCR array and sequencing experiments; further validation analyses may identify additional potential miRNA biomarkers of early pregnancy.

## Conclusions

Through RNA sequencing and qPCR profiling in cattle, we identified for the first time changes in the levels of miRNAs in plasma during early pregnancy. Specifically, we identified an increase (up to 2-fold) in the levels of miR-26a during Days 16 to 24 of pregnancy. These changes putatively reflect changes in miRNA expression in one or more body tissues and may play potentially important roles in the establishment of pregnancy. Overall, the differences in plasma miRNAs levels between pregnant and non-pregnant animals were small; larger differences in circulating miRNAs may occur at later stages of pregnancy. In addition, a limited ability to accurately quantify low abundance RNAs using available technologies may have prevented detecting larger differences in miRNA levels during early pregnancy. In summary, our results identify miR-26a as a novel candidate biomarker of early pregnancy in cattle.

## Methods

### Experimental design and sample collection

During March-April of 2013, 24 cycling Holstein-Friesian heifers (14–17 months old) were oestrus-synchronised using Eazi-Breed™ CIDR® Cattle Insert (1.38 g progesterone over 8 days; Zoetis, USA), Receptal® (0.02 mg buserelin on the day of CIDR insertion; MSD Animal Health, UK) and Estrumate® (0.5 mg cloprostenol 7 days after CIDR insertion; MSD Animal Health). After 48 h (Day 0) animals were either sham-inseminated (non-pregnant group; n = 8) or inseminated with frozen semen (pregnant group; n = 16). Animals were inseminated or sham-inseminated again 24 h later. Blood was collected on Days 0, 8, 16 from all animals. Additional samples were collected on Day 24 from the pregnant group. Pregnancy was confirmed twice on Day 35 and Day 60 by trans-rectal ultrasound. Five animals failed to become pregnant after insemination and were excluded from the study.

Blood was collected from all animals in 4 × 10 mL K2 EDTA Vacutainer tubes (Becton Dickinson, USA) by jugular venepuncture, using 18G needles (Becton Dickinson), and stored at 4 °C. Within 2 h of collection samples were centrifuged at 1,900 × g for 10 min at 4 °C to remove blood cells, and then again at 16,000 × g for 10 min at 4 °C to remove cellular debris and platelets. All plasma samples were immediately frozen at −80 °C. All heifers used in this study were kept under the same housing and feeding conditions in a single farm and all animal procedures were carried out under the UK Home Office Animals (Scientific Procedures) Act 1986 with approval by the Ethical Review Committee, University of Edinburgh.

### RNA extraction

RNA was extracted from 1.05 mL of plasma using TRIzol LS (Life Technologies, USA), following the manufacturer’s protocol. During the RNA extraction protocol, glycogen (180 μg; Sigma-Aldrich, USA) was added to each sample to facilitate visualisation of precipitated RNA, and an exogenous miRNA control, syn-cel-miR-39-3p (0.25 fmol; Qiagen, NL), was spiked-in to each sample. RNA was re-suspended in 30 μL of RNase-free water and used immediately or frozen at −80 °C.

### Illumina small-RNA sequencing and data analysis

Small-RNA libraries were prepared using the Illumina TruSeq small-RNA sample preparation kit (Illumina, USA) following the manufacturer’s protocol. Libraries were submitted to 36-base single-end sequencing using the Illumina HiSeq 2000 platform. Raw sequencing data were processed using the sRNAtoolbox software [53]. The bovine genome (UMD 3.1) was used as reference; trimmed and quality-controlled reads were mapped against bovine (primarily) and human (for homologue identification) mature miRNAs from miRBase (accessed 11/06/2014; [54]) allowing only one-nucleotide mismatches. After mapping, human and bovine miRNA read counts were merged and normalised to generate reads per million mapped (RPMM). MiRNAs detected with less than 25 RPMMs in more than 75 % of the samples in each of the experimental groups were excluded, keeping 178 miRNAs for further analysis. The average within-animal expression of each miRNA in the non-pregnant group was calculated (mean of Days 0, 8 and 16) and used for further analyses (NP group). Normalised expression levels (RPMMs) were log2 transformed before applying two-sample t-tests on the data, followed by an FDR adjustment of the *P* values using R programming. The transformed data were normally distributed as determined by the D’Agostino-Pearson omnibus and Shapiro-Wilk normality tests. Comparisons made were NP16 vs P16 (a direct comparison between non-pregnant and pregnant groups on Day 16), NP vs P16 and NP vs P24.

### Qiagen custom PCR arrays

We used commercial 384-well Custom PCR arrays (Qiagen) which covered a total of 377 unique human miRNAs, 308 of which were conserved in cow (2 nucleotide mismatch allowed). Three pools of 3 to 4 samples each, from each of Day 0, Day 8 and Day 16 for non-pregnant animals, and from Day 24 pregnant animals (12 pools in total) were analysed. cDNA (10 μL) was synthesised from 2 μL RNA sample using miScript II RT kit (Qiagen) in a Whatman-Biometra Thermocycler (Biometra, USA). The arrays were setup according to the manufacturer’s instructions and were run on the LightCycler 480 System (Roche, Switzerland). Data analysis was performed using Microsoft Excel and R programming. Raw Cq data were initially filtered to remove wells with non-specific amplification as identified by melting-curve analysis. Next, miRNAs which had Cq > 35 in more than 75 % of samples per experimental group were removed from the dataset. Cq-values were normalised using the global mean expression, which was calculated from the miRNAs which were detected in all of the sample pools. The mean expression across non-pregnant time-points (Days 0, 8 and 16) was used for analyses, as for the sequencing data, resulting in 3 data-points for analyses from each of pregnant and non-pregnant groups (NP vs P24). The statistical analysis of the transformed normalised data was performed as described for the sequencing data above.

### RT-qPCR validation of high-throughput data

Results of high-throughput analyses (small-RNA sequencing or PCR arrays) were validated by RT-qPCR on the same plasma samples used for sequencing (*n* = 8 heifers/group). cDNA was generated as described above and diluted for use in 10 μL qPCR reactions using Qiagen SYBR Green kits in an Agilent Mx3005P qPCR system (Agilent Technologies, USA). Raw fluorescence data were processed using Agilent MxPro software. A fluorescence threshold of 0.1 was set for all experiments. The amplification efficiency generally ranged between 85 % - 115 %, with R^2^ > 0.85. Cq-values and gene expression data were processed using Microsoft Excel and statistical analysis was performed using GraphPad Prism 6 (GraphPad Software, USA). Specifically, data were log2 transformed and tested for normality as described for the small-RNA sequencing data, and Dunn’s multiple comparison tests (non-parametric) were used to generate P values for the comparisons of miRNA levels between non-pregnant (NP) and pregnant groups (P16 or P24).

### Validation of early pregnancy miRNA profiles in an independent group of heifers

During November 2015, 16 cycling, 14–17 month old Holstein-Friesian heifers were oestrus-synchronised and inseminated as described above. Blood samples were collected on Days 0 (before insemination), 16 and 24 and processed for RT-qPCR analysis as described above. Samples were analysed for 11 animals confirmed pregnant on Day 35.

## Availability of supporting data

Data sets supporting the results of this article are included within the article and its additional files.

## Additional files

Additional file 1: Determining haemolysis of bovine plasma samples. Samples with or without visible haemolysis were analysed to determine A) the ΔCq between miR-451 and miR-23, and B) optical density at 414 nm (as a measure of oxyhaemoglobin levels). ΔCq (C) and optical density (D) values for all plasma samples (*n* = 46) used for small RNA-sequencing and PCR array profiling, showing the absence of detectable haemolysis. Mean ± SEM is shown. (PDF 109 kb) Additional file 2: Small-RNA sequencing data. Normalised expression data (reads per million mapped) and differential expression analysis results for bovine plasma miRNAs, generated using small-RNA sequencing. (XLSX 102 kb) Additional file 3: PCR array data. Normalised expression data and differential expression analysis results for bovine plasma miRNAs, generated using PCR arrays. (XLSX 58 kb) Additional file 4: Differences in miRNA levels validated by qPCR. List of differences (*P* < 0.05) in miRNA abundance obtained by PCR array or sequencing (NP16 vs P16, NP vs P16 and NP vs P24) that were selected for validation by RT-qPCR. Fold changes were calculated as pregnant/non-pregnant. (XLSX 11 kb)

## Abbreviations

Cq: quantification cycle
miRNA: microRNA
NP: non-pregnant
P: pregnant
RPMM: reads per million mapped
RT-qPCR: reverse transcription – quantitative polymerase chain reaction

## References

[1] Gardiner J (2015). Farming Statistics - Livestock Populations at 1 December 2014.

[2] College of Agriculture, Food and Rural Enterprise. Extra Profit from Improved Herd Fertility. Belfast: Department of Agriculture and Rural Development; 2005.

[3] College of Agriculture, Food and Rural Enterprise. The Cost of Extended Calving Intervals. Belfast: Department of Agriculture and Rural Development; 2005.

[4] Ha M, Kim VN (2014). Regulation of microRNA biogenesis. Nat Rev Mol Cell Biol.

[5] McBride D, Carre W, Sontakke SD, Hogg CO, Law A, Donadeu FX (2012). Identification of miRNAs associated with the follicular-luteal transition in the ruminant ovary. Reproduction.

[6] Sontakke SD, Mohammed BT, McNeilly AS, Donadeu FX (2014). Characterization of microRNAs differentially expressed during bovine follicle development. Reproduction.

[7] Wessels JM, Edwards AK, Khalaj K, Kridli RT, Bidarimath M, Tayade C (2013). The MicroRNAome of Pregnancy: Deciphering miRNA Networks at the Maternal-Fetal Interface. PLoS ONE.

[8] Kresowick JD, Devor EJ, Van Voorhis BJ, Leslie KK. MicroRNA-31 Is Significantly Elevated in Both Human Endometrium and Serum During the Window of Implantation: A Potential Biomarker for Optimum Receptivity. Biology of reproduction. 2014. doi:10.1095/biolreprod.113.116590. 10.1095/biolreprod.113.116590 PMC6322437 24855107

[9] Su L, Liu R, Cheng W, Zhu M, Li X, Zhao S (2014). Expression Patterns of MicroRNAs in Porcine Endometrium and Their Potential Roles in Embryo Implantation and Placentation. PLoS ONE.

[10] Forde N, Duffy GB, McGettigan PA, Browne JA, Mehta JP, Kelly AK (2012). Evidence for an early endometrial response to pregnancy in cattle: both dependent upon and independent of interferon tau. Physiol Genomics.

[11] Tesfaye D, Worku D, Rings F, Phatsara C, Tholen E, Schellander K (2009). Identification and expression profiling of microRNAs during bovine oocyte maturation using heterologous approach. Mol Reprod Dev.

[12] Su L, Zhao S, Zhu M, Yu M (2010). Differential expression of microRNAs in porcine placentas on days 30 and 90 of gestation. Reprod Fertil Dev.

[13] Doridot L, Miralles F, Barbaux S, Vaiman D (2013). Trophoblasts, invasion, and microRNA. Front Genet.

[14] Zhou Y, Gong W, Xiao J, Wu J, Pan L, Li X (2014). Transcriptomic analysis reveals key regulators of mammogenesis and the pregnancy-lactation cycle. Sci China Life Sci.

[15] . Moldovan L, Batte KE, Trgovcich J, Wisler J, Marsh CB, Piper M. Methodological challenges in utilizing miRNAs as circulating biomarkers. J Cellular and Molecular Med. 2014. doi:10.1111/jcmm.12236. 10.1111/jcmm.12236 PMC3943687 24533657

[16] Mitchell PS, Parkin RK, Kroh EM, Fritz BR, Wyman SK, Pogosova-Agadjanyan EL (2008). Circulating microRNAs as stable blood-based markers for cancer detection. Proc Natl Acad Sci U S A.

[17] Turchinovich A, Weiz L, Langheinz A, Burwinkel B (2011). Characterization of extracellular circulating microRNA. Nucleic Acids Res.

[18] Vickers KC, Palmisano BT, Shoucri BM, Shamburek RD, Remaley AT (2011). MicroRNAs are transported in plasma and delivered to recipient cells by high-density lipoproteins. Nat Cell Biol.

[19] Kosaka N, Iguchi H, Ochiya T (2010). Circulating microRNA in body fluid: a new potential biomarker for cancer diagnosis and prognosis. Cancer Sci.

[20] Creemers EE, Tijsen AJ, Pinto YM (2012). Circulating microRNAs: novel biomarkers and extracellular communicators in cardiovascular disease?. Circ Res.

[21] Chevillet JR, Lee I, Briggs HA, He Y, Wang K (2014). Issues and prospects of microRNA-based biomarkers in blood and other body fluids. Molecules.

[22] Williams Z, Ben-Dov IZ, Elias R, Mihailovic A, Brown M, Rosenwaks Z (2013). Comprehensive profiling of circulating microRNA via small RNA sequencing of cDNA libraries reveals biomarker potential and limitations. Proc Natl Acad Sci U S A.

[23] Prieto DMM, Markert UR (2011). MicroRNAs in pregnancy. J Reprod Immunol.

[24] Kotlabova K, Doucha J, Hromadnikova I (2011). Placental-specific microRNA in maternal circulation--identification of appropriate pregnancy-associated microRNAs with diagnostic potential. J Reprod Immunol.

[25] Miura K, Morisaki S, Abe S, Higashijima A, Hasegawa Y, Miura S (2014). Circulating levels of maternal plasma cell-free pregnancy-associated placenta-specific microRNAs are associated with placental weight. Placenta.

[26] Chim SS, Shing TK, Hung EC, Leung TY, Lau TK, Chiu RW (2008). Detection and characterization of placental microRNAs in maternal plasma. Clin Chem.

[27] Cleys ER, Halleran JL, McWhorter E, Hergenreder J, Enriquez VA, da Silveira JC (2014). Identification of microRNAs in exosomes isolated from serum and umbilical cord blood, as well as placentomes of gestational day 90 pregnant sheep. Mol Reprod Dev.

[28] Spornraft M, Kirchner B, Pfaffl MW, Riedmaier I. Comparison of the miRNome and piRNome of bovine blood and plasma by small RNA sequencing. Biotechnology letters. 2015. doi:10.1007/s10529-015-1788-2. 10.1007/s10529-015-1788-2 25700822

[29] Al-Soud WA, Jonsson LJ, Radstrom P (2000). Identification and characterization of immunoglobulin G in blood as a major inhibitor of diagnostic PCR. J Clin Microbiol.

[30] Kirschner MB, Kao SC, Edelman JJ, Armstrong NJ, Vallely MP, van Zandwijk N (2011). Haemolysis during sample preparation alters microRNA content of plasma. PLoS ONE.

[31] Pritchard CC, Kroh E, Wood B, Arroyo JD, Dougherty KJ, Miyaji MM (2012). Blood cell origin of circulating microRNAs: a cautionary note for cancer biomarker studies. Cancer Prev Res (Phila).

[32] Kirschner MB, van Zandwijk N, Reid G (2013). Cell-free microRNAs: potential biomarkers in need of standardized reporting. Front Genet.

[33] Balhara AK, Gupta M, Singh S, Mohanty AK, Singh I (2013). Early pregnancy diagnosis in bovines: current status and future directions. Sci World J.

[34] Spornraft M, Kirchner B, Haase B, Benes V, Pfaffl MW, Riedmaier I (2014). Optimization of extraction of circulating RNAs from plasma--enabling small RNA sequencing. PLoS ONE.

[35] Ple H, Landry P, Benham A, Coarfa C, Gunaratne PH, Provost P (2012). The repertoire and features of human platelet microRNAs. PLoS ONE.

[36] Haider BA, Baras AS, McCall MN, Hertel JA, Cornish TC, Halushka MK (2014). A Critical Evaluation of microRNA Biomarkers in Non-Neoplastic Disease. PLoS ONE.

[37] Willeit P, Zampetaki A, Dudek K, Kaudewitz D, King A, Kirkby NS (2013). Circulating MicroRNAs as Novel Biomarkers for Platelet Activation. Circ Res.

[38] Wang K, Yuan Y, Cho J-H, McClarty S, Baxter D, Galas DJ (2012). Comparing the MicroRNA Spectrum between Serum and Plasma. PLoS ONE.

[39] Sorefan K, Pais H, Hall AE, Kozomara A, Griffiths-Jones S, Moulton V (2012). Reducing ligation bias of small RNAs in libraries for next generation sequencing. Silence.

[40] van Dijk EL, Jaszczyszyn Y, Thermes C (2014). Library preparation methods for next-generation sequencing: tone down the bias. Exp Cell Res.

[41] Fuchs RT, Sun Z, Zhuang F, Robb GB (2015). Bias in ligation-based small RNA sequencing library construction is determined by adaptor and RNA structure. PLoS ONE.

[42] Coutinho LL, Matukumalli LK, Sonstegard TS, Van Tassell CP, Gasbarre LC, Capuco AV (2007). Discovery and profiling of bovine microRNAs from immune-related and embryonic tissues. Physiol Genomics.

[43] Landgraf P, Rusu M, Sheridan R, Sewer A, Iovino N, Aravin A (2007). A mammalian microRNA expression atlas based on small RNA library sequencing. Cell.

[44] Tripurani SK, Xiao C, Salem M, Yao J (2010). Cloning and analysis of fetal ovary microRNAs in cattle. Anim Reprod Sci.

[45] Huang J, Ju Z, Li Q, Hou Q, Wang C, Li J (2011). Solexa sequencing of novel and differentially expressed microRNAs in testicular and ovarian tissues in Holstein cattle. Int J Biol Sci.

[46] Witwer KW, Sisk JM, Gama L, Clements JE (2010). MicroRNA regulation of IFN-beta protein expression: rapid and sensitive modulation of the innate immune response. J Immunol.

[47] Krawczynski K, Najmula J, Bauersachs S, Kaczmarek MM. MicroRNAome of Porcine Conceptuses and Trophoblasts: Expression Profile of microRNAs and Their Potential to Regulate Genes Crucial for Establishment of Pregnancy. Biol Reprod. 2014. doi:10.1095/biolreprod.114.123588. 10.1095/biolreprod.114.123588 25472924

[48] Zhang XD, Zhang YH, Ling YH, Liu Y, Cao HG, Yin ZJ (2013). Characterization and differential expression of microRNAs in the ovaries of pregnant and non-pregnant goats (Capra hircus). BMC Genomics.

[49] Wu L, Zhou H, Lin H, Qi J, Zhu C, Gao Z (2012). Circulating microRNAs are elevated in plasma from severe preeclamptic pregnancies. Reproduction.

[50] Salilew-Wondim D, Ahmad I, Gebremedhn S, Sahadevan S, Hossain MD, Rings F (2014). The expression pattern of microRNAs in granulosa cells of subordinate and dominant follicles during the early luteal phase of the bovine estrous cycle. PLoS ONE.

[51] Li R, Zhang CL, Liao XX, Chen D, Wang WQ, Zhu YH (2015). Transcriptome microRNA profiling of bovine mammary glands infected with Staphylococcus aureus. Int J Mol Sci.

[52] Mestdagh P, Hartmann N, Baeriswyl L, Andreasen D, Bernard N, Chen C (2014). Evaluation of quantitative miRNA expression platforms in the microRNA quality control (miRQC) study. Nat Methods.

[53] Rueda A, Barturen G, Lebron R, Gomez-Martin C, Alganza A, Oliver JL (2015). sRNAtoolbox: an integrated collection of small RNA research tools. Nucleic Acids Res.

[54] Griffiths-Jones S (2006). miRBase: the microRNA sequence database. Methods Mol Biol.

