# Supplementary figures and images for: Circulating miRNA signatures of early pregnancy in cattle
Source: BMC Genomics. 2016 Mar 3;17:184. doi: 10.1186/s12864-016-2529-1 (PMC4778341; doi:10.1186/s12864-016-2529-1)

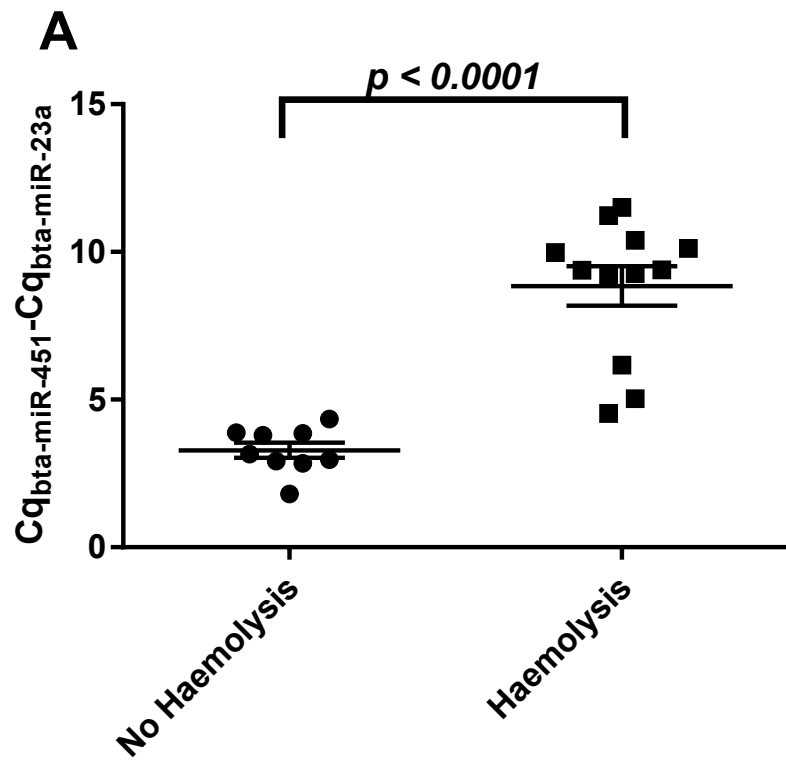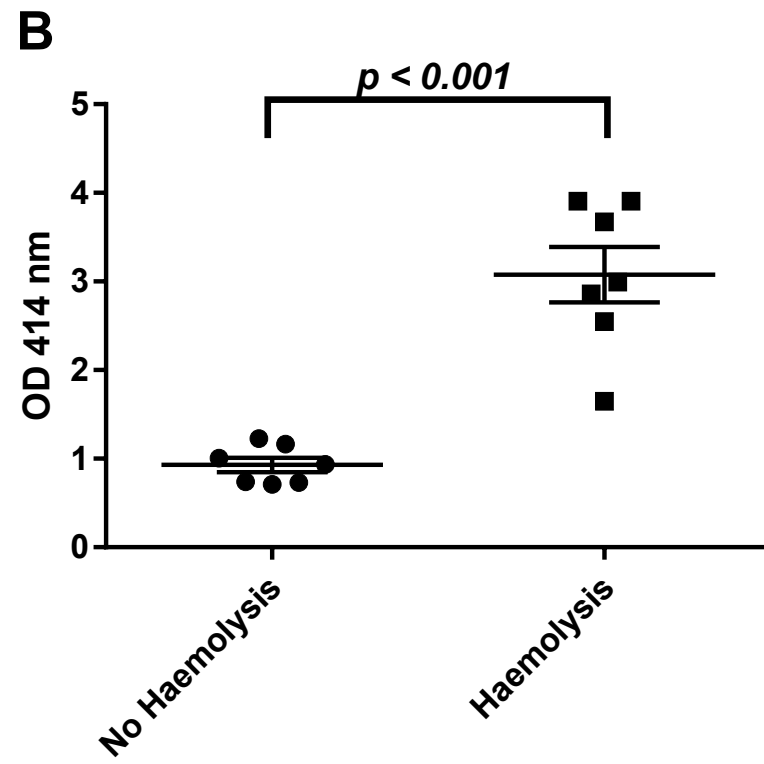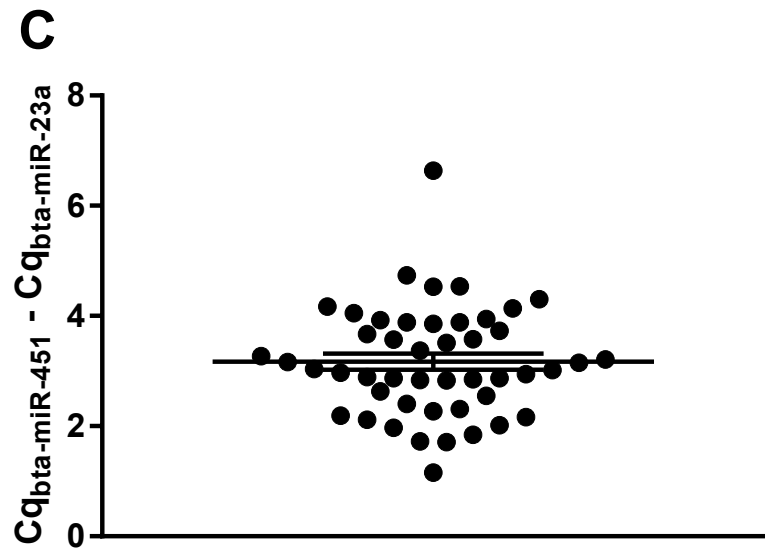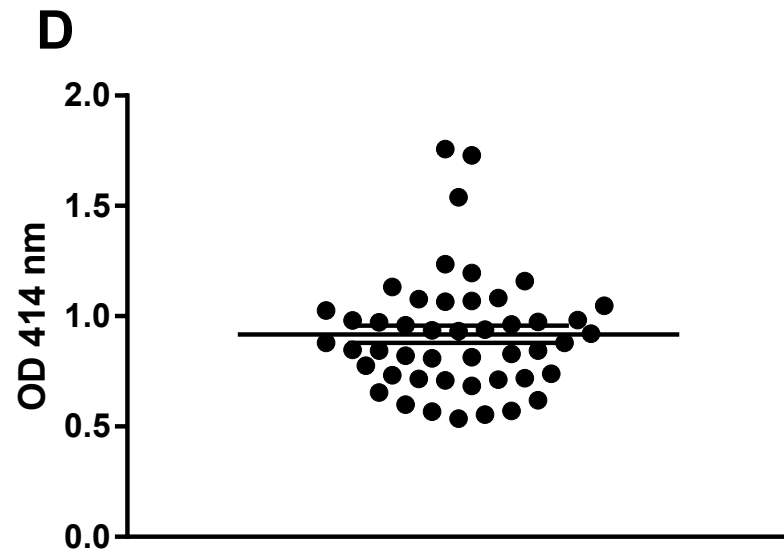

Supplement: Additional file 1: — Determining haemolysis of bovine plasma samples. Samples with or without visible haemolysis were analysed to determine A) the ΔCq between miR-451 and miR-23, and B) optical density at 414 nm (as a measure of oxyhaemoglobin levels). ΔCq (C) and optical density (D) values for all plasma samples (n = 46) used for small RNA-sequencing and PCR array profiling, showing the absence of detectable haemolysis. Mean ± SEM is shown. (PDF 109 kb) [file 12864_2016_2529_MOESM1_ESM.pdf]
